# A relationship to survival is seen by combining the factors of mismatch repair status, tumor location and age of onset in colorectal cancer patients

**DOI:** 10.1371/journal.pone.0172799

**Published:** 2017-03-02

**Authors:** Pan Li, Zhitao Xiao, Todd A. Braciak, Qingjian Ou, Gong Chen, Fuat S. Oduncu

**Affiliations:** 1 Department of Hematology and Oncology, Medizinische Klinik und Poliklinik IV, Ludwig Maximilians University, Munich, Germany; 2 Department of Colorectal Surgery, State Key Laboratory of Oncology in South China, Sun Yat-Sen University Cancer Center, Guangzhou, China; University of North Carolina at Chapel Hill School of Medicine, UNITED STATES

## Abstract

**Background:**

The progression of colorectal cancer (CRC) may differ depending on the location of the tumor and the age of onset of the disease. Previous studies also suggested that the molecular basis of CRC varies with tumor location, which could affect the clinical management of patients. Therefore, we performed survival analysis looking at different age groups and mismatch repair status (MMR) of CRC patients according to primary tumor location in an attempt to identify subgroups of CRC that might help in the prognosis of disease.

**Methods:**

A group of 2233 patients operated on to remove their CRC tumors were analyzed (521 with right colon cancer, 740 with left colon cancer and 972 with rectal cancer). The expression of four MMR genes was assessed by immunohistochemistry (IHC), independent of clinical criteria. From the data collected, a predictive model for overall survival (OS) could be constructed for some associations of tumor location and age of onset using Kaplan-Meier, logistic and Cox regression analysis.

**Results:**

When tumor location was considered as the lone factor, we found no statistical difference in overall survival (OS) between right cancer (68%), left cancer (67%) or rectal cancer tumor locations (71%) (HR: 1.17, 95%CI (confidence interval): 0.97–1.43, *P* = 0.057). When age of onset was considered, middle age (40–59 years) and older (60–85 years) patients were found to have higher OS than younger onset cancer (20–39 years) patients (69% vs 71% vs 59%, HR: 1.07, 95% confidence interval (CI): 0.91–1.25, *P* = 0.008). When both age of onset and tumor location were considered in combination as disease factors, we found that the subgroup of patients with left colon cancer from middle age (69%) and older (67%) aged patients had higher OS than younger (54%) patients (HR: 0.89, 95%CI: 0.68–1.16, *P* = 0.048). However in patients with right colon cancers, we found no statistical difference is OS between younger, middle age or older grouped patients (60% vs 71% vs 67%, HR: 0.84, 95% CI: 0.61–1.16, *P* = 0.194). With regard to rectal located cancers, we found that younger (62%) and middle age (68) patients had lower OS than older (77%) patients (HR:1.46, 95%CI: 1.13–1.88, *P* = 0.004). The rates of deficient MMR (dMMR) was 10.4%. We found no statistical difference in OS stratified by tumor locations. However, right colon cancer patients with dMMR (86%) had higher OS than those with proficient MMR (pMMR) (63%) (HR: 3.01, 95% CI: 1.82–4.97, *P*<0.001). Left colon cancer patients with dMMR (76%) also had higher OS than those with pMMR (66%) (HR: 1.67, 95% CI: 0.95–2.92, *P* = 0.01). Oppositely, rectal cancer patients with dMMR (60%) had lower OS than those pMMR (68%) (HR: 0.77, 95% CI: 0.51–1.17, *P* = 0.04).

**Conclusions:**

These data demonstrate that primary tumor location can be an important factor when considered along with age of onset for the prognosis of CRC. Primary tumor location is also an important factor to evaluate the predictive effect of MMR status for the prognosis of CRC.

## Introduction

It is estimated that colorectal cancer causes over 600,000 deaths worldwide annually, which makes this disease the fourth most common cause of cancer-related death[1,2]. Tumor location is now being considered to be an important factor for pathogenesis of CRC. Currently, noteworthy correlations between certain biological, clinical features and the anatomic subsites of the tumor have been made. Right colon cancers are more likely to be characterized by mucinous histology, high microsatellite instability, wild type p53 and BRAF mutation[3,4,5]. In contrast, left colon cancers are frequently found to be infiltrating, constricting lesions, with a phenotype that involves chromosomal instability[4]. While rectal located cancers are known to be more strongly associated with the presence of isolated pulmonary metastases than colon cancer [6]. While approximately 50% of the CRC occur in rectosigmoid area, a shift in location towards the proximal colon during the past few decades has been noted. The impact of this shift in CRC tumor location with regard to prognosis has not yet clearly been defined.

Another factor that has been considered that might have prognostic impact is the age of disease onset. It is known that the incidence of CRC increases markedly with advancing age. However, the overall notion that age is a significant prognostic factor in CRC has been controversial. Various studies have reported poorer prognosis among young patients with CRC[7]. While other reports have demonstrated that younger patients with CRC surgically treated to remove primary tumors appeared to have a higher specific survival rate than elderly patients [8,9].

CRCs with dMMR have distinct clinical and pathological features that commonly include proximal colon predominance, poor differentiation and increased numbers of tumor-infiltrating lynphonotes compared with CRCs with pMMR[10]. Most prior studies have suggested that right colon cancers are associated with higher mortality than left colon and rectally located cancers[11]. This finding would appear to be inconsistent with observations that right colon cancers are more likely to be dMMR[12]. To date, only a limited number of prospective studies have looked for a relationship between MMR status and prognosis of CRC by subsite across the colorectum[13,14]. Although many of these previous studies had large study populations, many case groups became small after stratification by MMR status and tumor site were used for analysis. While the dMMR phenotype has been reported to be up to 10-fold higher in frequency as seen in sporadic proximal compared to left tumors[15], there remains a need to better understand the epidemiologic and clinical profile of MMR status between right and left colon or rectally located cancers. Currently, only a limited number of prospective studies have evaluated the relation between tumor location and age of onset with regard to prognosis of CRC [16] [17].

To date, there has been no systematical analysis looking at the combined influence of MMR status, location and age of disease onset with regard to the prognosis of CRC. Therefore, we analyzed the relationship of CRC overall survival, MMR status and the age of onset stratified by tumor location using a large number of Chinese CRC patients whose disease outcomes were established.

## Methods

### Patients

The ethics committee of Sun Yat-Sen University Cancer Center approved this study and the written informed consents for all patients were obtained at the beginning of the study. A total of 4500 histologically confirmed patients with CRC were recruited after operation from Sun Yat-Sen University cancer center between May 2011 and May 2016. All patients were of Chinese origin. The clinical and family history for each of these patients was reviewed. Finally, 2233 cases were selected for analysis after application of the following strict exclusion criteria: patients age less than 18 years and older than 85 years, severe complication, multi-primary cancer, synchronous and metachronous CRC, family history (first-degree and second-degree relatives had any kind of cancer), familial adenomatous polyposis, death not due to tumor-related reason were not included in the study. The primary tumor location was categorized as right colon if the tumor was located above the splenic flexure or left colon if it was located at or below the splenic flexure and rectum. The median follow-up on living patients was 4.3 years.

### Treatment and follow-up

Stage I (T1–2 N0) and stage II (T3–4 N0) CRC patients without high-risk clinical features (e.g. T4 stage, bowel perforation or clinical bowel obstruction, inadequate lymph node sampling, poorly differentiated histology) were treated with radical surgery or endoscopic removal of the tumor alone. Stage II (T3–4 N0) CRC patients with high-risk clinical features were recommended to follow the XELODA/mFOLFOX/XELOX treatment regimen. Stage III (Tx N1–2) patients were scheduled to receive radical surgery and 12 cycles of adjuvant mFOLFOX/XELOX regimen treatment within a 6-month period. All stage IV (Tx Nx M1) patients received either palliative surgery or radical surgery. The first-line treatment for Stage IV CRC was the mFOLFOX/FOLFIRI chemotherapy regimen. Eighty-nine patients with rectal cancer also received neo-chemoradiotherapy. Responses were evaluated in accordance with the RECIST guidelines. After surgery, tumor recurrence was monitored by physical examination, serum carcinoembryonic antigen (CEA) assay, and abdominal and thoracic imaging taken every 3–6 months for the first 3 years following initial therapy, then every 6 months for the following 2 years, and finally from an annual check up. The duration of the patient follow-up was defined as the time between surgery and disease recurrence, death or last hospital contact (scheduled follow-up or telephone contact). The cutoff date for this analysis was May 2016.

### Immunohistochemistry

**Fig 1:**
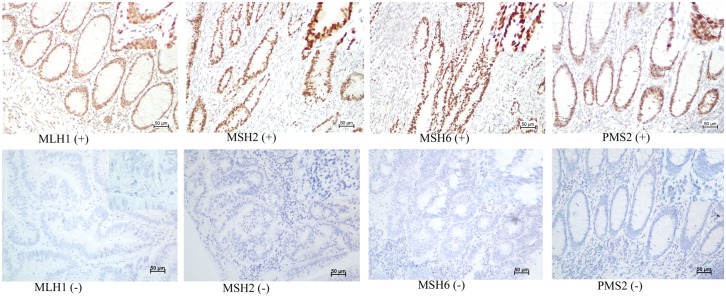
Illustrative immunostainings. Positive (upper panel) and negative (lower panel) for MLH1, MSH2, MSH6 and PMS2.

Blocks of formalin-fixed, paraffin-embedded adenocarcinoma tissue comprising an area of normal colorectal mucosa adjacent to the tumor were selected in each case. Cases with complete nuclear loss of expression in invasive tumor cells with retained expression in inflammatory cells and/or adjacent normal tissue as positive controls were considered MMR deficient. Staining was performed using the following primary antibodies: mouse anti-human mutL homolog 1 (MLH1) (dilution 1:150, clone OTI1C1, zhongshan jiqiao, Beijing), rabbit anti-human mutS homolog2 (MSH2) (dilution 1:100, clone ZA0622, zhongshan jiqiao, Beijing, mouse anti-human mutS homolog 6 (MSH6) (dilution 1:150, clone OTI5D1, zhongshan jiqiao, Beijing), and mouse anti-human postmeiotic segregation increased 2 (PMS2) (dilution 1:150, clone OTI2G5, zhongshan jiqiao, Beijing). Whole tissue sections were read separately by two pathologists blinded to the patients’ clinical characteristics. Discordant cases were reviewed by a supplementary pathologist to reach a consensus. Illustrative immunostainings are shown in Fig 1.

### Statistic analysis

Patient data were described as frequencies (percentages) in our analysis. Differences in distributions between the variables examined were assessed with the Χ^2^ or the Fisher’s exact test. The primary end point for this study was OS, defined as the time elapsed from the date of surgery until tumor-induced death. Surviving patients were censored on the last follow-up date. Median follow-up and the 95% CI were calculated using the reverse Kaplan–Meier method. The survival curve was estimated with the Kaplan–Meier method and compared using the log-rank test. Univariate and multivariable Cox proportional hazards models were used to explore the association of age, location, stage, differentiation grade and gender. The score and likelihood ratio test *P* values were used to test the statistical significance of each covariate in the univariate and multivariable Cox models, respectively. All statistical tests were two-sided, and *P* values less than or equal to 0.05 were considered statistically significant. Statistical analyses were performed using SPSS software.

## Results

The examined patient population included 521 (23.3%) right colon cancers, 740 (33.1%) left colon cancers and 972 (43.5%) rectal located cancers. A total of 2001 (89.6%) CRC specimens showed retained expression of MLH1, MSH2, MSH6 and PMS2 in tumor cells. In comparison, loss of expression in at least one of the four MMR genes occurred in only 232 of 2233 patients analyzed (10.4%). The results for demographics and tumor characteristics by location are listed in Table 1.

**Table 1:** Clinicopathological characteristics of patients.

Characteristic	Locations (n/%)			*P* value
	Right colon (521/23.3)	Left colon (740/33.1)	Rectum (972/43.5)	
**Gender**				0.164
**Male**	298 (13.3)	457 (20.5)	561 (25.1)	
**Female**	223 (10.0)	283 (12.7)	411 (18.4)	
**Age**				0.286
**20–39 years**	52 (2.3)	59 (2.6)	77 (3.4)	
**40–59 years**	243 (10.9)	323 (14.5)	458 (20.5)	
**60–85 years**	226 (10.1)	358 (16.0)	437 (19.6)	
**Pathology**				<0.001
**G1**	40 (1.8)	27 (1.2)	37 (1.7)	
**G2**	426 (19.1)	675 (30.2)	901 (40.3)	
**G3**	5 (0.2)	4 (0.2)	9 (0.4)	
**Mucinous**	45 (2.0)	27 (1.2)	23 (1.0)	
**Signet-ring**	5 (0.2)	7 (0.3)	2 (0.1)	
**Stage**				<0.001
**I**	27 (1.2)	74 (3.3)	230 (10.3)	
**IIA**	195 (8.7)	230 (10.3)	185 (8.3)	
**IIB**	23 (1.0)	36 (1.6)	166 (7.4)	
**IIC**	14 (0.6)	14 (0.6)	14 (0.6)	
**IIIA**	7 (0.3)	16 (0.7)	32 (1.4)	
**IIIB**	119 (5.3)	165 (7.4)	168 (7.5)	
**IIIC**	25 (1.1)	33 (1.5)	34 (1.5)	
**IVA**	63 (2.8)	98 (4.4)	104 (4.7)	
**IVB**	48 (2.1)	74 (3.3)	39 (1.7)	
**MMR status**				
**dMMR**	117 (5.2)	55 (2.5)	60 (2.7)	<0.001
**pMMR**	404 (18.1)	685 (30.7)	912 (40.8)	
**Alive**				0.057
**Yes**	355 (15.9)	494 (22.1)	695 (31.1)	
**No**	166 (7.4)	246 (11.0)	277 (12.4)	

In general, we found that colon cancers (77.1%) were more likely to be mucinous or signet-ring phenotype tumors than rectal cancers (22.9%) (*P*<0.001). The OS between the four stages of CRC showed significant differences (stage I: 94%, stage II: 83%, stage III: 70% and stage IV: 18%, HR 0.93, 95%CI: 0.65–2.38, *P*<0.001). With regard to tumor location alone, we found no statistical difference in OS between right (68%), left (67%) or rectal located cancers (71%) (HR: 1.17, 95%CI: 0.97–1.43, *P* = 0.057).

When age of disease onset was considered as a prognostic factor alone, we found that middle age (40–59 years) and older (60–85 years) patients had a statistically significant higher OS than younger (20–39 years) patients (69% vs 71% vs 59%, HR: 1.07, 95% CI: 0.91–1.25, *P* = 0.008). Additional stratification in patient prognosis profiling could be evidenced when age of disease onset was considered along with tumor location. When these factors were analyzed together, we found that left colon located cancers for both middle age (69%) and older (67%) patients had a statistically significant higher OS than that for younger (54%) patients (HR: 0.89, 95%CI: 0.68–1.16, *P* = 0.048). However this effect was not seen for right colon located cancers, in this case we found no statistical difference in OS between younger, middle age or older patients (60% vs 71% vs 67%, HR: 0.84, 95% CI: 0.61–1.16, *P* = 0.194). An effect was seen in rectal located cancers when age and tumor location were considered. Here, a statistically significant difference in survival was found for both younger (62%) and middle aged (68%) patients that had a lower OS than older (77%) patients (HR: 1.46, 95%CI: 1.13–1.88, *P* = 0.004).

When dMMR status was considered with location, right colon cancer patients with dMMR (86%) had higher OS than those patients with pMMR (63%) (HR: 3.01, 95% CI: 1.82–4.97, *P*<0.001). Likewise, left colon cancer patients with dMMR (76%) also had higher OS than those with pMMR (66%) (HR: 1.67, 95% CI: 0.95–2.92, *P* = 0.01). Oppositely, we found rectal cancer patients with dMMR (60%) had lower OS than those with pMMR (68%) (HR: 0.77, 95% CI: 0.51–1.17, *P* = 0.04).

**Fig 2:**
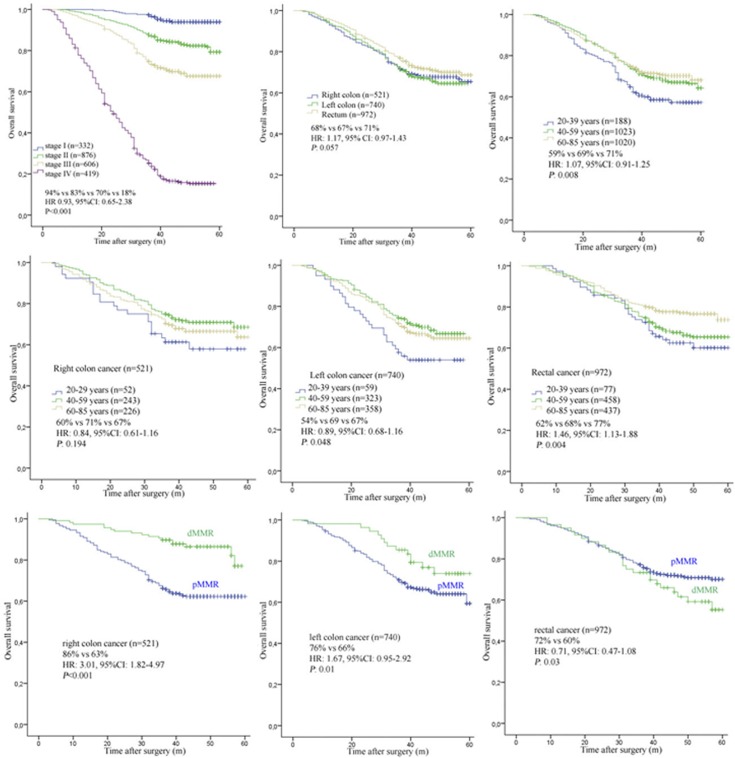
OS according to the MMR status, tumor location and age.

Among the variables analyzed in the multivariate Cox model, stage (HR 3.86, 95%CI: 3.69–4.01, *P* = 0.001), pathological differentiation (HR 2.14, 95%CI: 2.07–2.19, *P*<0.001) and MMR status (HR 1.23, 95%CI: 1.19–1.26, *P*<0.001) were significantly associated with tumor location. However, gender (HR 1.39, 95%CI: 1.35–1.42, *P* = 0.164) and the age of patients (HR 2.38, 95%CI: 2.33–2.42, *P* = 0.286) showed no statistical significance with tumor location. The survival plots of tumor location and age are shown in Fig 2.

## Discussion

Colorectal cancer has reported high morbidity and mortality rates worldwide, and according to the GLOBOCAN report from 2008, an estimated 1.2 million people suffered from CRC, accounting for approximately 10% of all cancer patients[18].

Various risk factors for CRC have been identified, and these form the basis for screening recommendations to be provided for patients. CRC, like other types of cancer, demonstrates an increasing incidence with age. In this current study, middle aged and older classified patients accounted for 45.9 and 45.7 percent of the CRC patient population studied with younger patients only accounting for the remaining 8.4% of the CRC cases. This patient distribution for CRC we found in our study was concordant with the patient breakdown seen in previous study[19].

The notion that age is a significant prognostic factor in CRC has previously been suggested but this conclusion has remained somewhat controversial. Previous studies have reported poorer prognosis among younger patients with CRC[7]. While others have reported that younger patients with CRC among those surgically treated appeared to have a higher specific survival rate versus more elderly patients [8,9]. In our report here on Chinese CRC patients, we found middle and older aged patients had higher OS than younger patients (*P* = 0.008). Our observation that cancer related mortality did not decrease with increasing age after those reaching 50 years old exemplifies the idea that, although elderly patients have a shorter life expectancy based on their age, still benefited from cancer treatment.

Another factor that we wanted to test that might be useful as a prognostic factor in CRC is the location of the primary CRC tumor. Different locations within the colon are derived from different embryonic tissues. The right colon (cecum, ascending colon, and proximal two thirds of the transverse colon) is derived from the embryonic midgut, whereas the left colon (distal one third of the transverse colon, descending colon, sigmoid colon, and rectum) is derived from the embryonic hindgut[20]. It is likely that these different origins of colon tissue could contribute to CRC disease pathology by independent processes. Indeed, there is already evidence to indicate that the clinical characteristics and molecular profiles of gene and protein expression for CRC differ across these different tissue sites[21,22,23,24]. One previous study has already shown that the primary tumor location could serve as prognostic factor in metastatic CRC[25]. Other studies reported about some correlation between patients’ sex and tumor location [26,27]. However, in this study we found no statistical association between sex and tumor location. In this study, we found no statistical difference in OS between right cancer, left cancer and rectal cancer (*P* = 0.057). It is not yet clear why we did not find a similar result when analyzing tumor location alone independent of age of disease onset. Yet, when tumor location was considered along with age of onset, we could find statistically significant associations for tumor location to disease survival.

Tumor location and age of onset are two basic clinical characteristics of CRC. To date, only a limited number of prospective studies have evaluated the relation between tumor location and age of onset to the prognosis of CRC. It has been previously reported that right-sided early-onset colon cancers were a subset in which most Lynch Syndrome cases could be found, with earlier stages of disease at diagnosis having better prognosis[16]. In another study, the Bax/Bcl-2 ratio was statistically correlated for CRC against age and tumor location. Here, patients older than 50 showed decreased levels of Bax/Bcl-2 ratio. Moreover, with regard to tumor location, the Bax/Bcl-2 ratio was found to be significantly lower in colon compared to rectal cancer[17]. However, one potential caveat to these findings is that both studies only focused on a select age group of cancer patients.

In our current study, we found two possibly important associations for tumor location and age of disease onset to OS of CRC patients. First in left colon located cancers, middle (69%) and older aged (67%) patients were found to have higher OS than younger (54%) patients (*P* = 0.048). Second in rectal located cancers, we found the younger (62%) and middle aged (68) patients had lower OS than older (77%) patients (*P* = 0.004). According to the well-accepted model of CRC progression by Vogelstein, malignancy arises with aging as a result of accumulation of mutations in tumor suppressor genes and oncogenes [28,29]. Because we have noticed differences in OS with regard to tumor location and not necessarily due to age, our results suggest that another factor in disease could be from accumulation effect of mutations that differ in various tissue locations. Recently, it was reported that human intestinal microbiota and bacterial metabolites contributed to the aetiology of CRC[30]. It is likely that possible mechanisms are involved and gaining understanding of these processes will aid in the prognosis of CRC patients.

From our analysis we were able to find that more frequent occurrences of mucinous, high-grade and signet-ring phenotype in right colon cancers (9.6%) compared with left colon cancer (4.6%) and rectal cancer (2.6%). It is of note that right lesions have more frequently been reported to be related to dMMR status tumors. One explanation for this finding is that the right and left colon arise from different embryological sources [31].

In clinical practice, the tumor's MMR status is increasingly being used to guide clinical management. Stage II patients with dMMR have a better prognosis and may actually be harmed by 5-FU treatment[32]. We found that patients with right tumors had no prognostic difference compared with patients who had left colon and rectal tumors, which contradicted to the previous findings of others[33,34]. Yet in our current study, we did find that dMMR status could show benefit as a prognostic biomarker for right and left colon cancer. As well, dMMR gave indications for bad prognosis of rectal cancers. Overall we found that the separate evaluation of tumor location appears to have some predictive and prognostic value when combined with MMR status for colon and rectal cancers.

## Conclusions

We have found that the combination of age and tumor location can be a significant predictor of overall survival in CRC. This finding solidifies the notion that despite the morbidity and mortality associated with any CRC diagnosis, baseline patient characteristics might still provide predictive information as to a given patient’s outcome and also can help the clinician to predict the clinical course and response of chemotherapy. Our findings also confirm the importance of location of primary tumor and indicate that tumors resected from different locations of the colorectum have different dMMR status that can work as prognostic biomarkers in colorectal cancer.

The increasing trend in the CRC mortality indicate that improved primary and secondary prevention measures are particularly still needed and could be further aided by better prognostic methods being made available.
